# Total Ionizing Dose Effects on the Threshold Voltage of GaN Cascode Devices

**DOI:** 10.3390/mi14101832

**Published:** 2023-09-26

**Authors:** Hao Wu, Xiaojun Fu, Jun Luo, Manlin Yang, Xiaoyu Yang, Wei Huang, Huan Zhang, Fan Xiang, Yang Pu, Ziwei Wang

**Affiliations:** 1 National Key Laboratory of Integrated Circuits and Microsystems, Chongqing 401332, China; xjfu2000@163.com (X.F.); manlin_yang62@163.com (M.Y.); yxy19910411@163.com (X.Y.); puyang7227@163.com (Y.P.); ziweiwangs@outlook.com (Z.W.); 2 No. 24 Research Institute of China Electronics Technology Group Corporation, Chongqing 401332, China; ncdaluo2000@163.com (J.L.); huangweifirst@163.com (W.H.); 18693053653@163.com (H.Z.); xiangfanx@163.com (F.X.)

**Keywords:** GaN MISHEMT device, Cascode device, total ionizing dose effect, threshold voltage shift

## Abstract

GaN devices are nowadays attracting global attention due to their outstanding performance in high voltage, high frequency, and anti-radiation ability. Research on total ionizing dose and annealing effects on E-mode GaN Cascode devices has been carried out. The Cascode device consists of a low-voltage MOSFET and a high-voltage depletion-mode GaN MISHEMT. Cascode devices of both conventional processed MOSFET and radiation-hardened MOSFET devices are fabricated to observe the TID effects. Experiment results indicate that, for the Cascode device with conventional processed MOSFET, the *V*_TH_ shifts to negative values at 100 krad(Si). For the Cascode device with radiation-hardened MOSFET, the *V*_TH_ shifts by −0.5 V at 100 krad(Si), while shifts to negative values are 500 krad(Si). The annealing process, after the TID experiment, shows that it can release trapped charges and help *V*_TH_ recover. On one hand, the *V*_TH_ shift and recover trends are similar to those of a single MOSFET device, suggesting that the MOSFET is the vulnerable part in the Cascode which determines the anti-TID ability of the device. On the other hand, the *V*_TH_ shift amount of the Cascode device is much larger than that of a previously reported p-GaN HEMT device, indicating that GaN material shows a better anti-TID ability than Si.

## 1. Introduction

For decades of development, silicon-based MOSFET devices have reached their ability limits [1]. With the rapidly increased demands of power electronics, novel materials, such as GaN and SiC, are nowadays becoming more and more popular due to their capabilities of high power, temperature, frequency, and radiation tolerance [2,3,4]. GaN devices are widely considered to be excellent candidates for microwave use [5,6,7,8,9,10,11,12,13,14]. Recently, GaN materials have also become promising candidates for power use due to their advantages of high voltage and rapid switching time [15]. Nowadays, enhancement-mode (E-mode), or the normally-off p-GaN gate HEMTs, have become the most commercial type as its threshold voltage is above 0 V, where the safety of the circuit is guaranteed. However, low-gate breakdown voltage [16], low-threshold voltage [17], and current-collapse effect [18] are still restricting the direct replacement of MOSFET. Especially, the gate-to-source breakdown voltage of p-GaN gate HEMTs is normally 6 V, while the conventional output of the driver chip in the power system is usually 0~10 V. At present, Cascode devices can be the temporary substitution for its ability of high-gate breakdown voltage and high-threshold voltage, despite the fact that the switching time is slower due to the existence of Si MOSFET.

In this study, we fabricated high-voltage Cascode devices combined with depletion-mode (D-mode) GaN MISHEMT (Metal-Insulator-Semiconductor High Electron Mobility Transistor) and conventional processed MOSFET or radiation-hardened MOSFET. The total ionizing dose (TID) effect of the Cascode device is analyzed, and comparisons between both types of devices are made. As monitoring the negative shift of the threshold voltage of the MOSFET is well known and the standard process under TID-irradiation, we found out that Cascode devices share a similar phenomenon.

## 2. Device Structure and Test Results

**Figure 1:**
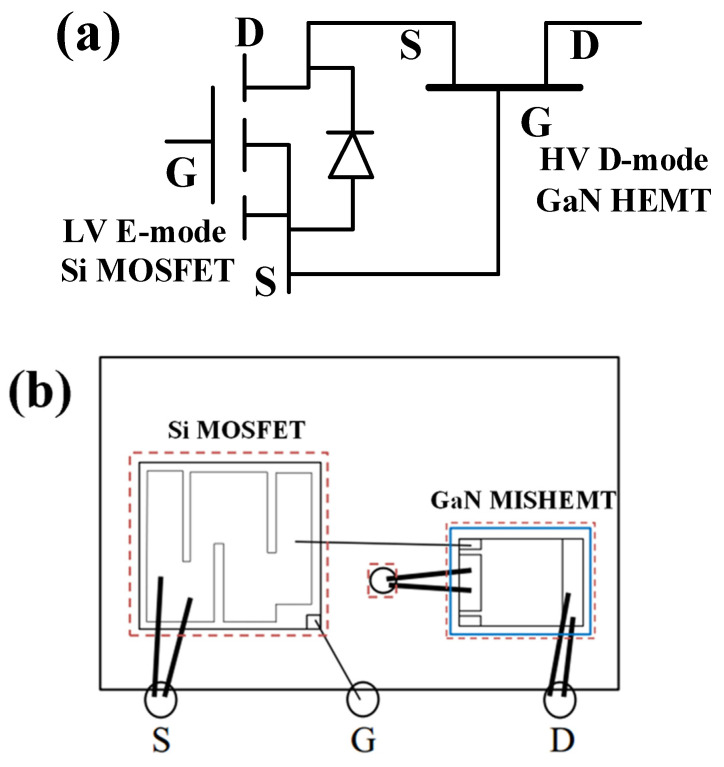
The schematic diagram (**a**) and the package (**b**) of the Cascode device.

A Cascode device combines a high-voltage D-mode GaN MISHEMT device and a low-voltage E-mode Si MOSFET. The high-voltage GaN MISHEMT brings high drain-to-source breakdown voltage. While the low-voltage MOSFET brings high gate breakdown voltage, due to the existence of SiO_2_, which, on the other hand, leads to more severe TID effects. The schematic diagram and the package of the Cascode device are shown in Figure 1.

As shown in Figure 1a, as a whole, the source of the MOSFET is the source of the Cascode device. The gate of the MOSFET is the gate of the Cascode device, and the drain of the GaN HEMT device is the drain of the Cascode device. Inside the Cascode device, as shown in Figure 1b, the back of the MOSFET is the drain, which is soldered to the bottom of the packaging case. The source of the MOSFET is connected to the gate of the GaN MISHEMT device. The source of the GaN MISHEMT device is connected to the bottom of the device, which is also connected to the drain of the MOSFET. The MOSFET device chip size is 3.4 mm × 3.3 mm, and the GaN MISHEMT device size is 2.3 mm × 1.8 mm.

To better understand the influences of the TID effects, two types of Cascode devices are prepared. Type A device consists of a conventional processed (not radiation-hardened) MOSFET device and a D-mode GaN MISHEMT device, while Type B device consists of a radiation-hardened MOSFET devices and a D-mode GaN MISHEMT device in the same chip size.

For the radiation-hardened MOSFET devices, there are several special processes to enhance the anti-TID ability, as we reported before [19].

**Figure 2:**
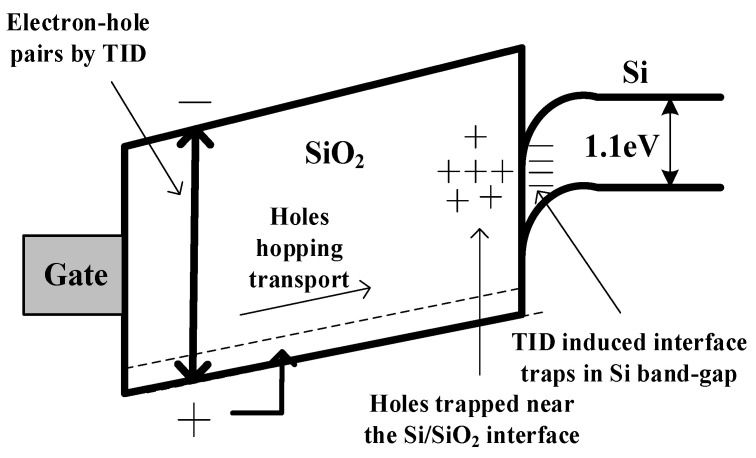
TID effect of MOSFET.

(1) Gate oxide growth atmosphere control. The charged defects of the gate oxide layer of MOSFETs include two parts: charges introduced by manufacturing processes and charges introduced by TID irradiation, as shown in Figure 2. To obtain a better quality dielectric layer, we arrange the gate oxide growth process after the high-temperature drive-in process to prevent the possible impurities in the silicon from diffusing into the gate oxide layer during the high-temperature drive-in process, which might bring more defects. Also, we add a proper amount of impurities, such as chlorine gas, during the oxidation process to change the stress distribution of the local valence bond at the interface, reducing the local stress of the gate dielectric layer. We also use Ar instead of N_2_ as the annealing gas during the oxide growth annealing process to reduce the concentration of Si-H and Si-OH bonds.

(2) Optimization of body region implantation. The TID-induced positive charges near the Si/SiO_2_ interface can easily cause gate leakage current. Therefore, the body region implantation conditions are optimized to reduce this leakage. On one hand, traditional one-time field implantation is changed to multiple implantations, thus, the ion concentration of the body region edge is improved, depressing the influence of the interface positive charges caused by TID radiation. On the other hand, the implantation dose is optimized.

(3) Thin gate oxide thickness. The TID effect is strongly correlated with the gate oxide layer thickness. Generally, the thinner gate oxide thickness obtains smaller charges. Therefore, the gate oxide thickness, which is 100 Å~200 Å thinner than the conventional process MOSFET, is designed.

For the fabricated D-mode GaN HEMT devices, a metal-insulator-semiconductor (MIS) structure is designed to depress the gate current leak and, thus, improve the device’s reliability [20].

**Figure 3:**
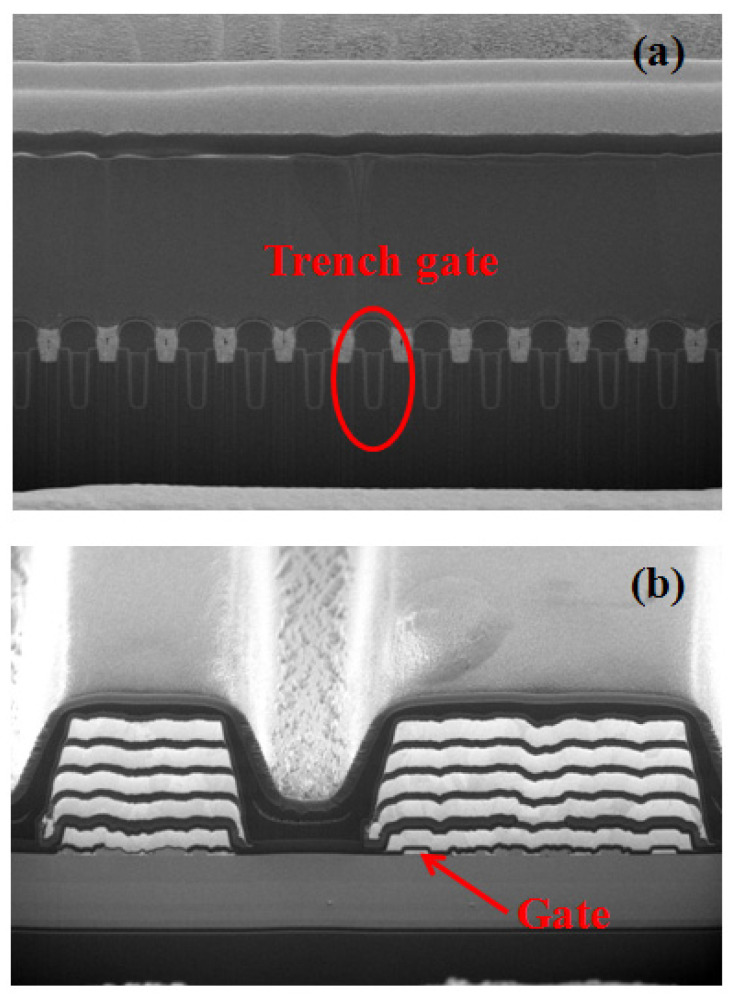
Microscopic views of the fabricated devices, where (**a**) is the trench gate MOSFET and (**b**) is the GaN MISHEMT.

Static parameters of the breakdown voltage, threshold voltage, and on-state resistance are tested for both types of devices. The breakdown voltage (*BV*) of Type A devices is 710 V, the threshold voltage (*V*_TH_) is +3.0 V, and the on-state resistance (*R*_ON_) is 0.13 Ω. The *BV* of Type B devices is 710 V, the *V*_TH_ is +3.6 V, and the *R*_ON_ is 0.13 Ω. The microscopic views of the fabricated devices are shown in Figure 3, where Figure 3a is the trench gate MOSFET and Figure 3b is the GaN MISHEMT.

## 3. TID Experiments and Annealing Effects

### 3.1. TID Experiments

**Figure 4:**
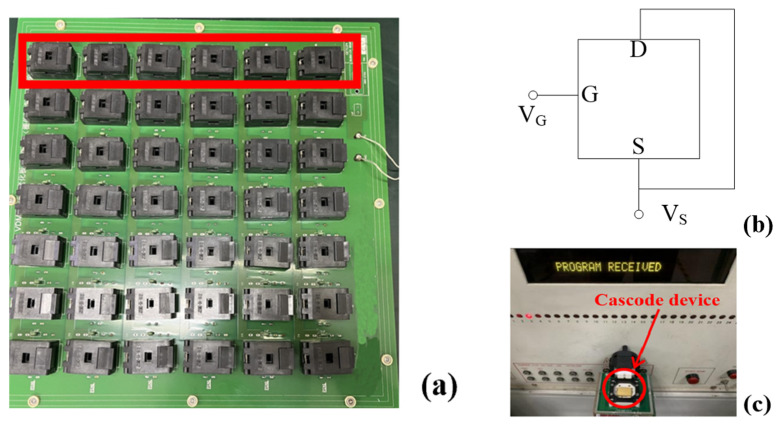
TID experiment board. Each six devices is connected parallelly as a group (**a**). Irradiation configurations of individual devices (**b**). BR3500 semiconductor discrete device tester to test the device’s parameters (**c**).

We prepared 24 devices, of which 12 were Type A and 12 were Type B. Each six devices was connected parallelly as a group, the drain and source electrodes were shorted and jointly led out, and the gate electrodes were led out separately, as shown in Figure 4a. The irradiation configurations of the individual device are shown in Figure 4b. We took the TID experiments at the Institute of Nuclear Physics and Chemistry, Chinese Academy of Engineering Physics. Both types of devices underwent TID experiments. The devices were irradiated at a 50 rad(Si)/s dose rate and a total dose of 500 krad(Si) under Co-60 conditions. After every 50 krad(Si) dose, the devices were tested once. In this research, “bias mode” is used during the irradiation process, and the gate bias voltage applied to the devices was set to be +12 V, which is the common work bias condition. We used a BR3500 semiconductor discrete device tester to test the device’s parameters before and after the TID and annealing processes, as shown in Figure 4c. BV is tested at *I*_D_ = 1.0 mA, *V*_GS_ = 0 V, *V*_th_ is tested at *I*_D_ = 1.0 mA, *V*_DS_ = *V*_GS_, and *R*_ON_ is tested at *V*_GS_ = 10 V, *I*_D_ = 5 A. All the parameters are tested at *T*_A_ = 25 °C. At higher temperatures, we used NTH64-70A to provide a stable high-temperature annealing environment. The presented data are calculated by the average test results.

#### 3.1.1. Type A Devices

Since no TID-hardened measures were taken, the threshold voltage of the Type A devices shifted to negative values at 100 krad(Si). Although other static characteristics of the devices, such as *BV* and *R*_ON,_ were still normal (measured under the condition of *V*_GS_ < 0 V), the devices were regarded as failed because they were no longer controllable and would seriously threaten the safety of the system. The pre- and post-TID results of the Type A devices are shown in Table 1. The tested results show that the *V*_TH_ of the device shifted from +3.0 V to +1.2 V at 50 krad(Si), and the shift amount was −1.8 V. At 100 krad(Si), the *V*_TH_ shifted to below 0 V, and the shift amount exceeded −3 V. This trend is consistent with the *V*_TH_ shift trend of a single MOSFET device. For the *BV* of the device, there was almost no change, indicating that the total dose effect had a very small impact on the depletion-mode GaN MISHEMT device within 100 krad(Si). Since the device had failed at 100 krad(Si), the experiment stopped and no higher doses were added.

#### 3.1.2. Type B Devices

**Figure 5:**
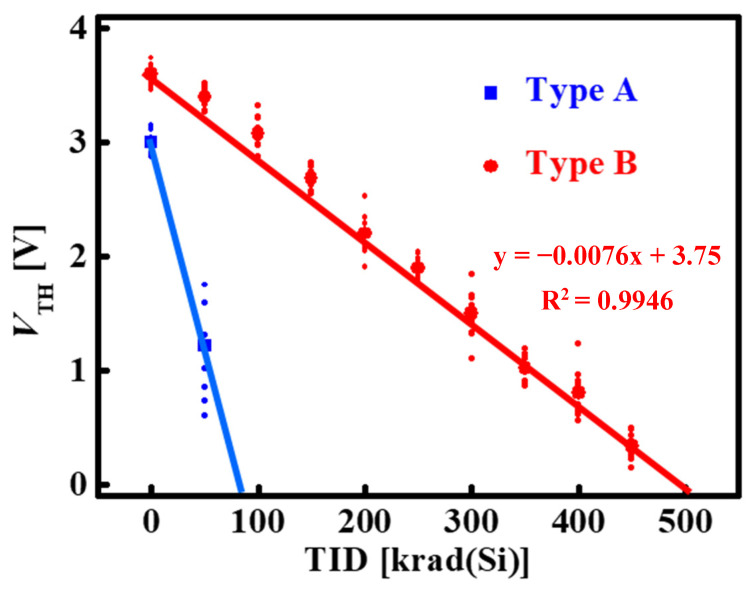
The threshold voltages of Type A and Type B devices at different total doses. The small dots are the tested values, while the large dots are the calculated average values. The red line is the linear fitting for Type B, and the blue line is the linear fitting for Type A. The calculated slope is −0.0076 mV/krad(Si). The goodness of fits (R^2^) is 0.9946, which is higher than 0.99, showing good linear fitting.

For the devices with radiation-hardened measures, the *V*_TH_ of the Type B devices also shifted negatively (TID dose, which shifted to +3.1 V at 100 krad(Si), and the shift amount was −0.5 V). By adding higher doses, it was found that the *V*_TH_ of the device shifted to negative values at 500 krad(Si). The experimental results are presented in Table 2, where for *BV* and *R*_ON_, the calculated average results are shown, while for *V*_TH_, all the tested individual results are shown, and the standard deviations under each dose amount and the linear fitted lines in Figure 5 are calculated. The standard deviation of each group of devices ranges from 0.072 to 0.214, which shows a relatively good consistency for our experiment. The threshold voltage shift trend of the device was similar to that of the radiation-hardened MOSFET device as we reported before [19], indicating that the MOSFET device is the key factor limiting the total dose capability of the Cascode devices. The *BV* of the device slightly decreases and the *R*_ON_ slightly increases at higher doses, due to the high dose rate which leads to the decrease of the two-dimensional electron gas concentration in the device, which is consistent with the research results of X. Sun [21] and Y. S. Puzyrev [22]. The threshold voltages of Type A and Type B devices at different doses are shown in Figure 5.

From the tested results above, it is observed that, compared with D-mode GaN MISHEMT devices, Si MOSFET devices are easier to fail in TID experiments, regardless of whether TID-radiation hardening measures are taken or not. This phenomenon indicates that the anti-TID ability of the GaN MISHEMT device is stronger than that of the MOSFET device, even when the MOSFET device has been radiation hardened. Overall, since the total ionizing dose level of 100 krad(Si) is sufficient for utilization in aerospace, Cascode devices with radiation-hardened measures can be a potential candidate in general aerospace systems.

Previously, we reported the TID and annealing effects of p-GaN HEMT devices [3]. The largest threshold voltage shift amount is −0.2 V at 500 krad(Si), which is significantly smaller than the Cascode devices with/without radiation-hardened MOSFET in this research. This result indicates that p-GaN devices show better anti-TID ability than Cascode devices. The unstable *V*_TH_ of the power device may cause difficulties in the design of the front-stage driver or controller.

### 3.2. Annealing Process

**Figure 6:**
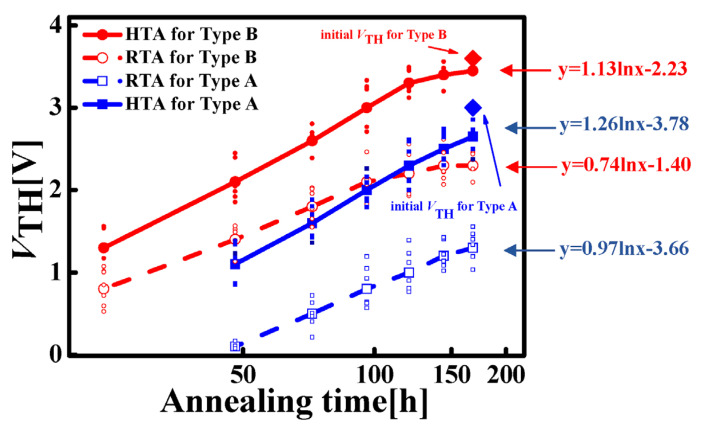
Recovery of *V*_TH_ of both types. The red solid dots are the tested values of HTA for Type B. The red hollow dots are the tested values of RTA for Type B. The blue solid dots are the tested values of HTA for Type A. The blue hollow dots are the tested values of RTA for Type A. The solid and dashed lines are the calculated average values. The formulas on the right side are the fitted recovery trends.

After the TID experiments, the annealing processes were taken to observe the recovery of *V*_TH_. The received TID-radiation doses of the Type A and Type B devices are 100 krad(Si) and 500 krad(Si), respectively. A 168 h high-temperature annealing (HTA, *T*_A_ = 100 °C) and a 168 h room temperature annealing (RTA, *T*_A_ = 25 °C) were separately taken, and the recovery of *V*_TH_ was observed. During the annealing process, the *V*_TH_ of the device was tested every 12 h. The experiment results are shown in Figure 6.

As annealing has been well considered a popular method for repairing TID-induced SiO_2_ damages [23], tested results show that for both types of devices, the *V*_TH_ gradually recovered from negative to positive, indicating that the TID effect did not cause complete damage to the device, and the *V*_TH_ is partially recoverable. The annealing process releases the trapped charges, leading to the recombination of electrons and holes, and, thus, reduces the amount of trap charges both at the interface of Si/SiO_2_ and inside SiO_2_ of the MOSFET device. Therefore, the *V*_TH_ is recovered, where the *V*_TH_ of Type A under RTA returns to an average value of 1.32 V, the *V*_TH_ of Type A under HTA returns to 2.65 V, the *V*_TH_ of Type B under RTA returns to 2.32 V, and the *V*_TH_ of Type B under HTA returns to 3.45 V. It is observed that both Type A and Type B devices show faster recovery trends of *V*_TH_ under HTA, as high temperature can accelerate the release process of trapped charges. However, it is also observed that the *V*_TH_ could not fully recover to the initial value, where the *V*_TH_ of Type A under RTA remains a −1.7 V gap, the *V*_TH_ of Type A under HTA remains a −0.35 V gap, the *V*_TH_ of Type B under RTA remains a −1.3 V gap, and the *V*_TH_ of Type B under HTA remains a −0.3 V gap. The gaps between the annealed device and the original device are partly due to the lack of energy to allow all trapped charges to be fully released. On the other hand, some electrons were swept out of the device during the total dose experiment processes, resulting in a lower total electron concentration. The annealing processes all follow exponential recovery with time (hours). The fitting slopes in Figure 6 of HTA for Type A and B are 1.26 V/h and 1.136 V/h, while those of RTA for Type A and B are 0.97 V/h and 0.74 V/h. Their results indicate that under HTA, the *V*_TH_ shifts faster than that under RTA. P. J. McWhorter et al. [23] show that for MOSFETs, the recovery slope at *T*_A_ = 25 °C is about 0.026 V/h, which is much smaller compared with our work. We suggest that the large difference is due to the fact that we fabricated the devices in our experimental processes, thus the trap amount is much larger than that of commercial devices.

## 4. Conclusions

In this work, we designed Cascode devices combined with low-voltage MOSFET and high-voltage D-mode GaN MISHEMT. Both the conventional processed MOSFET device and the radiation-hardened MOSFET device were fabricated to analyze the TID effects. Experiment results show that the threshold voltage of the Cascode device shifts negatively, of which the shift trench is similar to a single MOSFET device, indicating that the MOSFET device is the key limiting the anti-radiation ability of the Cascode device, and annealing processes after the TID process help the threshold voltage recover. Compared with the p-GaN HEMT device, the Cascode device shows a more sensitive response to TID. While the advantages of high-gate breakdown voltage and high threshold voltage make Cascode a better device for directly substituting traditional high-voltage MOSFET. We believe that p-GaN HEMT devices and Cascode devices will share great properties in separate fields of power applications.

## Figures and Tables

**Table 1:** Pre- and post-TID results of Type A (average from the tested results).

	*BV*	*V*_th_	*R*_ON_
Pre-TID	710 V	+3.0 V	0.13 Ω
50 krad(Si)	710 V	+1.2 V	0.13 Ω
100 krad(Si)	-	<0 V	-

**Table 2:** Pre- and post-TID results of Type B (*BV* and *R*_ON_ are the averages from the tested results).

Dose/rad(Si)	*BV*/V	*V*_th_/V								*R*_ON_/Ω
		Individual Device						Std	Ave	
Pre-TID	710	3.525	3.623	3.486	3.690	3.743	3.598	0.072	3.6	0.13
		3.598	3.612	3.546	3.662	3.686	3.558			
50 k	710	3.340	3.446	3.285	3.267	3.499	3.524	0.082	3.4	0.13
		3.382	3.396	3.343	3.480	3.496	3.374			
100 k	710	2.986	3.074	2.980	2.876	3.227	3.328	0.122	3.1	0.13
		3.050	3.058	2.994	3.129	3.220	3.027			
150 k	710	2.576	2.725	2.556	2.552	2.794	2.827	0.089	2.7	0.14
		2.681	2.723	2.628	2.753	2.755	2.665			
200 k	710	2.085	2.242	2.052	1.909	2.345	2.533	0.149	2.2	0.14
		2.189	2.198	2.175	2.242	2.296	2.182			
250 k	710	1.820	1.901	1.187	1.794	2.033	2.047	0.214	1.9	0.15
		1.896	1.896	1.824	1.952	1.983	1.834			
300 k	710	1.341	1.57	1.319	1.107	1.658	1.850	0.110	1.5	0.15
		1.481	1.522	1.415	1.644	1.647	1.433			
350 k	710	0.906	1.11	0.878	0.870	1.153	1.191	0.180	1.0	0.15
		1.032	1.052	0.913	1.112	1.133	0.985			
400 k	708	0.644	0.858	0.616	0.563	0.968	1.236	0.134	0.8	0.16
		0.799	0.846	0.650	0.892	0.910	0.703			
450 k	708	0.249	0.388	0.223	0.150	0.486	0.496	0.072	0.3	0.16
		0.323	0.358	0.275	0.429	0.430	0.286			
500 k	-	<0								-

Note: “std” stands for “standard deviation”, and “ave” stands for “average”.

## Data Availability

Data available on request due to restrictions e.g., privacy or ethical.

## References

[1] Rupp R., Laska T., Haberlen O., Treu M. (2014). Application specific trade-offs for WBG SiC, GaN and high end si power switch technologies. IEDM Tech. Dig..

[2] Chen K.J., Hberlen O., Lidow A., Tsai C.L., Ueda T., Uemoto Y., Wu Y. (2017). GaN-on-Si power technology: Devices and applications. IEEE Trans. Electron Devices.

[3] Wu H., Fu X., Guo J., Liu T., Wang Y., Luo J., Huang Z., Hu S. (2022). Total ionizing dose and annealing effects on *V*_TH_ shift for p-GaN Gate AlGaN/GaN MISHEMTs. IEEE Electron Device Lett..

[4] Wang Y., Lin M., Li X., Wu X., Yang J., Bao M., Yu C., Cao F. (2019). Single-Event Burnout Hardness for the 4H-SiCTrench-Gate MOSFETs Based on the Multi-Island Buffer Layer. IEEE Trans. Electron Devices.

[5] Hariya A., Koga T., Matsuura K., Yanagi H., Tomioka S., Ishizuka Y., Ninomiya T. (2017). Circuit design techniques for reducing the effects of magnetic flux on GaN-HEMTs in 5-MHz 100-W high power-density LLC resonant DC-DC converters. IEEE Trans. Power Electron..

[6] Wei J., Xie R., Xu H., Wang H., Wang Y., Hua M., Zhong K., Tang G., He J., Zhang M. (2019). Charge Storage Mechanism of Drain Induced Dynamic Threshold Voltage Shift in p-GaN Gate HEMTs. IEEE Trans. Electron Devices.

[7] Wu H., Fu X., Guo J., Wang Y., Liu T., Hu S. (2022). Time-Resolved Threshold Voltage Instability of 650-V Schottky Type p-GaN Gate HEMT Under Temperature-Dependent Forward and Reverse Gate Bias Conditions. IEEE Trans. Electron Devices.

[8] Shi Y., Shao D., Feng W., Zhang J., Zhou M. (2019). Silicon Interposer Package for MMIC Heterogeneous Integration Based on Gold/Solder Ball Flip-Chip Technique. IEEE Trans. Compon. Packag. Manuf. Technol..

[9] Udabe A., Baraia-Etxaburu I., Diez D.G. (2023). Gallium Nitride Power Devices: A State of the Art Review. IEEE Access.

[10] Ajayan J., Nirmal D., Mohankumar P., Mounika B., Bhattacharya S., Tayal S., Augustine A.S. (2022). Fletcher Challenges in material processing and reliability issues in AlGaN/GaN HEMTs on silicon wafers for future RF power electronics & switching applications: A critical review. Mater. Sci. Semicond. Process..

[11] Kozak J.P., Zhang R., Porter M., Song Q., Liu J., Wang B., Wang R., Saito W., Zhang Y. (2023). Stability, Reliability, and Robustness of GaN Power Devices: A Review. IEEE Trans. Power Electron..

[12] Mounika B., Ajayan J., Bhattacharya S., Nirmal D. (2022). Recent developments in materials, architectures and processing of AlGaN/GaN HEMTs for future RF and power electronic applications: A critical review. Micro Nanostruct..

[13] Zhang H., Sun Y., Hu K., Yang L., Liang K., Xing Z., Wang H., Zhang M., Yu H., Fang S. (2023). Boosted high-temperature electrical characteristics of AlGaN/GaN HEMTs with rationally designed compositionally graded AlGaN back barriers. Sci. China Inf. Sci..

[14] Sun Y., Zhang H., Yang L., Hu K., Xing Z., Liang K., Yu H., Fang S., Kang Y., Wang D. (2022). Correlation Between Electrical Performance and Gate Width of GaN-Based HEMTs. IEEE Electron Device Lett..

[15] Huang S., Wang X., Liu X., Li Y., Fan J., Yin H., Wei K., Zheng Y., Sun Q., Shen B. (2021). Interface Charge Effects on 2-D Electron Gas in Vertical-Scaled Ultrathin-Barrier AlGaN/GaN Heterostructure. IEEE Trans. Electron Devices.

[16] Zhang L., Zheng Z., Yang S., Song W., He J., Chen K.J. (2021). p-GaN gate HEMT with surface reinforcement for enhanced gate reliability. IEEE Electron Device Lett..

[17] Tang X., Li B., Moghadam H.A., Tanner P., Han J., Dimitrijev S. (2018). Mechanism of threshold voltage shift in p-GaN gate AlGaN/GaN transistor. IEEE Electron Device Lett..

[18] Kawanago T., Kakushima K., Kataoka Y., Nishiyama A., Sugii N., Wakabayashi H., Tsutsui K., Natori K., Iwai H. (2014). Gate technology contributions to collapse of drain current in AlGaN/GaN Schottky HEMT. IEEE Trans. Electron. Devices.

[19] Yang F., Wu H., Fu X., Xiang F., Xiang F. A Radiation-Hardened Trench Power MOSFET for Aerospace Applications. Proceedings of the 2018 IEEE Asia Pacific Conference on Circuits and Systems (APCCAS).

[20] Oka T., Nozawa T. (2008). AlGaN/GaN recessed MIS-gate HFET with high-threshold voltage normally-off operation for power electronics applications. IEEE Electron Device Lett..

[21] Sun X., Saadat O.I., Chen J., Zhang E.X., Cui S., Palacios T., Fleetwood D.M., Ma T.P. (2013). Total-Ionizing-Dose Radiation Effects in AlGaN/GaN HEMTs and MOS-HEMTs. IEEE Trans. Nucl. Sci..

[22] Puzyrev Y.S., Roy T., Zhang E.X., Fleetwood D.M., Schrimpf R.D., Pantelides S.T. (2011). Radiation-Induced Defect Evolution and Electrical Degradation of AlGaN/GaN High-Electron-Mobility Transistors. IEEE Trans. Nucl. Sci..

[23] McWhorter P.J., Miller S.L., Miller W.M. (1990). Modeling the anneal of radiation-induced trapped holes in a varying thermal environment. IEEE Trans. Nucl. Sci..

